# Supplementary material for: Isoform-resolved mRNA profiling of ribosome load defines interplay of HIF and mTOR dysregulation in kidney cancer
Source: Nat Struct Mol Biol. 2022 Sep 12;29(9):871–80. doi: 10.1038/s41594-022-00819-2 (PMC9507966; doi:10.1038/s41594-022-00819-2)
Supplement: Supplementary file 1 — Supplementary analyses, discussion and notes [file 41594_2022_819_MOESM1_ESM.pdf]

nature portfolio

https://doi.org/10.1038/s41594-022-00819-2

Supplementary information

Isoform-resolved mRNA profiling of ribosome load defines interplay of HIF and mTOR dysregulation in kidney cancer

In the format provided by the authors and unedited

Supplementary Information

Discussion of methods to study mRNA translation in a genome-wide manner

High-throughput DNA sequencing-based methods have been used to study translation of mRNAs in a genome-wide manner[18]. The two major methods for this purpose are ribosome profiling and polysome profiling[18,22]. These methods have complementary advantages. Ribosome profiling digests mRNA regions which are not occupied by the ribosome, and analyses the ribosome footprints using high-throughput DNA sequencing. By comparing the number of ribosome footprint reads with that of mRNA-Seq reads from the CDS of same mRNAs, the method estimates translational efficiency. It has an advantage in identifying the exact position of the ribosome at a specific time point. This enables the identification of the coding regions of mRNAs. In addition, since the footprint length changes in accordance with conformational changes during the elongation cycle, the method can also provide some information on translational elongation rate[84,85]. Ribosome profiling is probably the most-widely used method to study translation in a genome-wide manner. However, it is challenging for ribosome profiling to resolve mRNA isoforms, and the estimated translation efficiency will be affected by global changes in translation and/or transcription due to the difficulty in combining it with the use of external RNA standards[22]. In addition, the relatively short length of ribosome footprints, and large proportion of cDNAs from ribosomal RNAs[86] in the library can limit the complexity of the data.

Polysome profiling fractionates mRNAs by the number of associated ribosomes using a sucrose density gradient. To evaluate translational efficiency of mRNAs, polysome profiling-based methods typically compare pooled mRNAs from the fractions bound by a large number of ribosomes (e.g. > 3 ribosomes) with those bound by a small number of ribosomes or total mRNA. Since polysome profiling retains the integrity of mRNAs during fractionation, it has been also used to resolve differences in translation of mRNA isoforms by alternative splicing[20] or transcription state sites (TSS)[19,23]. In addition, in principle the method can be integrated with the use of external RNA standards, although to date this has not commonly been done. Polysome profiling has been successfully applied to study changes in translation such as by hypoxia[5–7] and mTOR inhibition[24,32,33]. However, unlike ribosome profiling, the polysome profiling cannot locate the position of the ribosome on an mRNA. Thus, the method is not able to distinguish translation of uORFs from the main CDS nor detect changes in elongation rate.

HP5, which is based on polysome profiling, was developed to provide a high-throughput method capable of comparing changes in translation across multiple conditions and combining this with resolution of transcripts by their TSS usage. The combination of high-resolution polysome profiling and external normalization of data enables the accurate measurement of the mean ribosome load of mRNAs. This is particularly useful when comparing translational efficiency of mRNAs across conditions since the measurements will not be confounded by global changes in translation and/or transcription[5,22]. Furthermore, the HP5 protocol generates cDNA libraries with a longer sequence reads and a greater proportion of informative reads derived from mRNAs compared to ribosome profiling.

Validation of the mean ribosome load (MRL) calculation from 1-8+ ribosome fraction

In the main report, mRNAs which are not loaded at all (‘0’ ribosome fraction) were not included in the analysis. This was because of the technical challenge of collecting this fraction accurately. To test whether the omission of the ‘0’ ribosome fraction affects the MRL calculation, we repeated certain analyses including this fraction. Data in the principal components analysis shows that the mRNA content of the ‘0’ ribosome fraction was similar to that of the ‘1’ ribosome fraction (Supplementary Fig. a). Furthermore, the values of MRL were very similar when calculated with or without including ‘0’ ribosome fraction (Supplementary Fig. b). Consideration of ‘0’ ribosome fraction also has little effect on the calculation of the changes in translation upon Torin 1 treatment (Supplementary Fig. c). These results demonstrate that omission of ‘0’ ribosome fraction had a negligible effect on the analysis of translation under the conditions of our experiments.

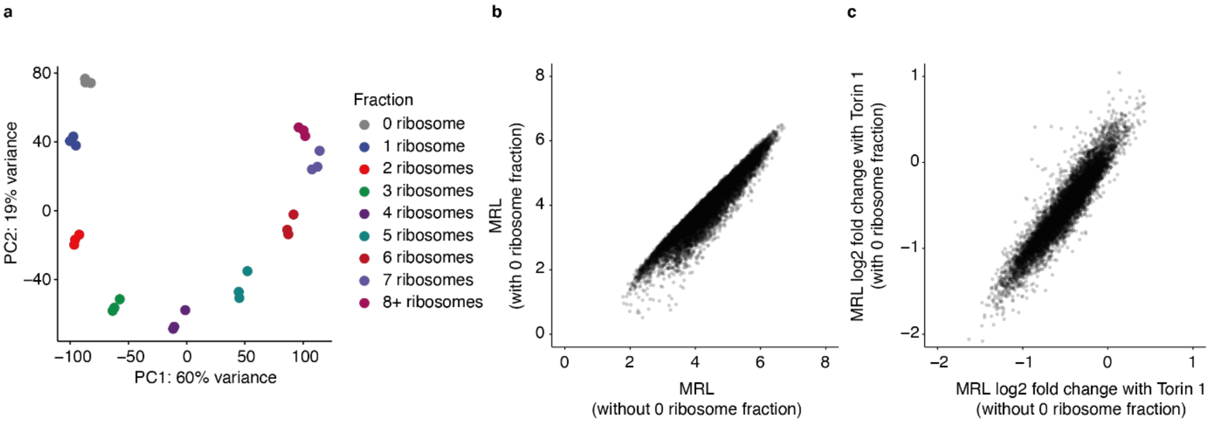

Supplementary Fig.
(a) Principal component analysis of HP5 data by polysome fraction. The data is identical to Fig. 1b in the main manuscript, but the analysis includes mRNAs from ‘0’ ribosome fractions. **(b)** Scatter plot comparing the mean ribosome load (MRL) for each gene in RCC4 VHL cells calculated with and without inclusion of mRNAs in the ‘0’ ribosome fraction. **(c)** Scatter plot comparing the changes in translational efficiency, expressed as log2 fold change in MRL for each gene with Torin 1, calculated with and without inclusion of mRNAs in the ‘0’ ribosome fraction. Note in **b**, the sharp demarcation above the line of identity is created because inclusion of any value for the ‘0’ ribosome fraction cannot increase MRL.

Supplementary Notes

Summary of exact n and *p* values for the analyses displayed in figures.

Fig. 1
(d)

CDS length	n	*p*
(100, 178]	41	< 10E-10
(178, 316]	468	< 10E-10
(316, 562]	1,344	< 10E-10
(562, 1000]	3,082	< 10E-3
(1000, 1778]	4,100	Reference
(1778, 3162]	2,309	< 10E-10
(3162, 5623]	731	< 10E-10
(5623, 10000]	162	< 10E-10
(10000, 17783]	30	< 10E-5

(e)

uORF number	n	*p*
0	6,794	Reference
1	2,706	< 10E-10
2	1,325	< 10E-10
3+	1,443	< 10E-10

(f)

mRNA feature	n	*p*
uORF number	445	<10E-6
RNA structure (near cap)	945	<10E-6
Kozak consensus	161	0.01

Fig. 2
(c)

Functional class	*p*
Transcription factors	<10E-10
Transcription machinery	1.0
Messenger RNA biogenesis	1.0
Spliceosome	1.0
Cytoplasmic ribosome	<10E-10
Mitochondrial ribosome	<10E-10
Translation factors	<10E-4

Chaperones and folding catalysts	<10E-6
Membrane trafficking	0.2
Ubiquitin system	<10E-6
Proteasome	<10E-10
Glycolysis	<10E-6
Pentose phosphate pathway	<10E-6
TCA cycle	0.002
Fatty acid biosynthesis	0.01
Fatty acid degradation	0.01
Oxphos	<10E-5
Nucleotide metabolism	<10E-4
Amino acid metabolism	<10E-4

(d)

Start position	Length	n	*p*
1	0	6,883	Reference
1	1	1,835	<10E-10
1	2	449	<10E-10
1	3	178	<10E-10
1	4	101	<10E-10
1	5	53	<10E-10
1	6	36	<10E-10
1	7	22	<10E-4
1	8+	32	<10E-10
2	0	3,507	Reference
2	1	4,675	0.03
2	2	1,066	<10E-10
2	3	240	<10E-3
2	4	72	1.0
2	5	17	1.0
2	6	7	1.0
2	7	3	1.0
2	8+	2	1.0
3	0	6,731	Reference
3	1	2,488	1.0
3	2	276	1.0
3	3	64	1.0
3	4	22	1.0
3	5	4	1.0
3	6	3	1.0
3	7	1	1.0

3	8+	0	NA

(e)

Treatment	uORF number	n	*p*
No treatment	0	5,635	Reference
No treatment	1	2,037	<10E-10
No treatment	2	948	<10E-10
No treatment	3+	969	<10E-10
Torin 1	0	5,635	Reference
Torin 1	1	2,037	<10E-3
Torin 1	2	948	0.04
Torin 1	3+	969	<10E-6

(f)

Treatment	CDS length	n
No treatment	(100, 178]	27
No treatment	(178, 316]	376
No treatment	(316, 562]	1,087
No treatment	(562, 1000]	2,461
No treatment	(1000, 1778]	3,143
No treatment	(1778, 3162]	1,791
No treatment	(3162, 5623]	556
No treatment	(5623, 10000]	126
No treatment	(10000, 17783]	22
Torin 1	(100, 178]	27
Torin 1	(178, 316]	376
Torin 1	(316, 562]	1,087
Torin 1	(562, 1000]	2,461
Torin 1	(1000, 1778]	3,143
Torin 1	(1778, 3162]	1,791
Torin 1	(3162, 5623]	556
Torin 1	(5623, 10000]	126
Torin 1	(10000, 17783]	22

Fig. 3-5 and Extended Data Fig. 3-10

Mean ribosome load for each condition was calculated as the combined average of the biological replicate data:

n = 3 (RCC4, RCC4 VHL, 786-O and 786-VHL)

n = 2 (RCC4 with Torin 1, RCC4 VHL with Torin 1, 786-O (*EIF4E2* KO; one clone was generated using g1 gRNA and a second using g2 gRNA), and 786-O VHL (*EIF4E2* KO; one clone was generated using g1 gRNA and a second using g2 gRNA)

mRNA abundance for each condition was calculated as the combined average of the biological replicate data:
n = 3 (RCC4 and RCC4 VHL)
n = 4 (786-O and 786-O VHL)

Fig. 3
(e)

Cell	TOP motif length	n	*p*
RCC4	0	8,206	Reference
RCC4	1	2,211	<10E-10
RCC4	2	532	<10E-10
RCC4	3	213	<10E-10
RCC4	4	120	<10E-10
RCC4	5	63	<10E-10
RCC4	6	38	<10E-10
RCC4	7	29	<10E-5
RCC4	8+	35	<10E-10
786-O	0	5,902	Reference
786-O	1	1,723	1.0
786-O	2	374	1.0
786-O	3	135	1.0
786-O	4	105	1.0
786-O	5	46	1.0
786-O	6	30	1.0
786-O	7	21	1.0
786-O	8+	27	1.0

Extended Data Fig. 3
(b)
Significance of mRNA features to predict mean ribosome load

Segment	RNA feature	n	*p*
5' UTR	Length	12,268	<10E-10
5' UTR	uORF number	12,268	<10E-10
5' UTR	RNA structure (near cap)	12,266	<10E-10
5' UTR	RNA structure (distal)	7,325	<10E-6
CDS	Kozak consensus	12,268	<10E-10
CDS	Length	12,268	<10E-10

CDS	RNA structure	12,268	<10E-10

Calculation of R^2 (only n for iteration 1 is shown as a representative data)

Iteration	Segment	RNA feature	n (training)	n (test)
1	5' UTR	Length	9,469	1,457
1	5' UTR	uORF number	9,469	1,457
1	5' UTR	RNA structure (near cap)	9,468	1,457
1	5' UTR	RNA structure (distal)	5,607	766
1	CDS	Kozak consensus	9,469	1,457
1	CDS	Length	9,469	1,457
1	CDS	RNA structure	9,469	1,457

(c)

MFE (-kcal/mol/nt)	n	*p*
[0.0064, 0.278]	2,454	Reference
(0.278, 0.344]	2,454	Reference
(0.344, 0.402]	2,450	Reference
(0.402, 0.474]	2,454	Reference
(0.474, 1.1]	2,454	<10E-10

(d)

Kozak consensus score	n	*p*
[0.164,0.56]	2,454	<10E-10
(0.56,0.642]	2,454	0.002
(0.642,0.712]	2,453	Reference
(0.712,0.785]	2,453	0.03
(0.785,0.989]	2,454	0.02

Extended Data Fig. 4
(b)

Data	mTOR targets defined by	n	*p*
HP5	(all)	9,461	Reference
HP5	Hsieh *et al.*	134	<10E-10
HP5	Thoreen *et al.*	205	<10E-10
HP5	Larsson *et al.*	315	<10E-10
HP5	Morita et al.	18	<10E-3
Hsieh *et al.*	(all)	6,607	Reference
Hsieh *et al.*	Hsieh *et al.*	136	NA

Hsieh *et al.*	Thoreen *et al.*	185	<10E-10
Hsieh *et al.*	Larsson *et al.*	238	0.03
Hsieh *et al.*	Morita et al.	13	0.5
Thoreen *et al.*	(all)	4,528	Reference
Thoreen *et al.*	Hsieh *et al.*	67	<10E-10
Thoreen *et al.*	Thoreen *et al.*	219	NA
Thoreen *et al.*	Larsson *et al.*	193	0.02
Thoreen *et al.*	Morita et al.	7	1.0

Extended Data Fig. 5
(b)

CDS length	n	*p*
(100, 178]	27	<10E-4
(178, 316]	376	<10E-10
(316, 562]	1,087	<10E-3
(562, 1000]	2,461	<10E-10
(1000, 1778]	3,143	<10E-10
(1778, 3162]	1,791	<10E-10
(3162, 5623]	556	<10E-10
(5623, 10000]	126	<10E-10
(10000, 17783]	22	<10E-9

(c)

Treatment	Transcript length	n
No treatment	(178, 316]	1
No treatment	(316, 562]	103
No treatment	(562, 1000]	521
No treatment	(1000, 1778]	1636
No treatment	(1778, 3162]	2994
No treatment	(3162, 5623]	2718
No treatment	(5623, 10000]	1326
No treatment	(10000, 17783]	265
No treatment	(17783, 31623]	24
No treatment	(31623, 56234]	1
Torin 1	(178, 316]	1
Torin 1	(316, 562]	103
Torin 1	(562, 1000]	521
Torin 1	(1000, 1778]	1636
Torin 1	(1778, 3162]	2994
Torin 1	(3162, 5623]	2718

Torin 1	(5623, 10000]	1326
Torin 1	(10000, 17783]	265
Torin 1	(17783, 31623]	24
Torin 1	(31623, 56234]	1

(d)

Panel	ΔMRL log2 fold change	n	*p*
TOP motif length	Small (1 ~ 1.2)	1,327	<10E-10
TOP motif length	Medium (1.2 ~ 1.5)	627	<10E-10
TOP motif length	Large (> 1.5)	159	<10E-10
uORF number	Small (1 ~ 1.2)	572	<10E-3
uORF number	Medium (1.2 ~ 1.5)	302	<10E-10
uORF number	Large (> 1.5)	92	<10E-7

References

84. Lareau, L. F., Hite, D. H., Hogan, G. J. & Brown, P. O. Distinct stages of the translation elongation cycle revealed by sequencing ribosome-protected mRNA fragments. *eLife* 2014, 1–16 (2014).

85. Wu, C. C. C., Zinshteyn, B., Wehner, K. A. & Green, R. High-Resolution Ribosome Profiling Defines Discrete Ribosome Elongation States and Translational Regulation during Cellular Stress. *Molecular Cell* 73, 959-970.e5 (2019).

86. Zinshteyn, B., Wangen, J. R., Boyang, H. U. A. & Green, R. Nuclease-mediated depletion biases in ribosome footprint profiling libraries. *RNA* 26, 1481–1488 (2020).
