# Supplementary material for: Isoform-resolved mRNA profiling of ribosome load defines interplay of HIF and mTOR dysregulation in kidney cancer
Source: Nat Struct Mol Biol. 2022 Sep 12;29(9):871–80. doi: 10.1038/s41594-022-00819-2 (PMC9507966; doi:10.1038/s41594-022-00819-2)
Supplement: Supplementary file 2 — Reporting Summary [file 41594_2022_819_MOESM2_ESM.pdf]

nature research

Corresponding author(s): Peter J Ratcliffe

Last updated by author(s): May 26, 2022

Reporting Summary

Nature Research wishes to improve the reproducibility of the work that we publish. This form provides structure for consistency and transparency in reporting. For further information on Nature Research policies, see our Editorial Policies and the Editorial Policy Checklist.

Statistics

For all statistical analyses, confirm that the following items are present in the figure legend, table legend, main text, or Methods section.

n/a	Confirmed	
☐	☒	The exact sample size (*n*) for each experimental group/condition, given as a discrete number and unit of measurement
☐	☒	A statement on whether measurements were taken from distinct samples or whether the same sample was measured repeatedly
☐	☒	The statistical test(s) used AND whether they are one- or two-sided<br>*Only common tests should be described solely by name; describe more complex techniques in the Methods section.*
☒	☐	A description of all covariates tested
☐	☒	A description of any assumptions or corrections, such as tests of normality and adjustment for multiple comparisons
☐	☒	A full description of the statistical parameters including central tendency (e.g. means) or other basic estimates (e.g. regression coefficient) AND variation (e.g. standard deviation) or associated estimates of uncertainty (e.g. confidence intervals)
☐	☒	For null hypothesis testing, the test statistic (e.g. *F*, *t*, *r*) with confidence intervals, effect sizes, degrees of freedom and *P* value noted<br>*Give P values as exact values whenever suitable.*
☒	☐	For Bayesian analysis, information on the choice of priors and Markov chain Monte Carlo settings
☒	☐	For hierarchical and complex designs, identification of the appropriate level for tests and full reporting of outcomes
☐	☒	Estimates of effect sizes (e.g. Cohen's *d*, Pearson's *r*), indicating how they were calculated

Our web collection on statistics for biologists contains articles on many of the points above.

Software and code

Policy information about availability of computer code

Data collection	High-throughput DNA sequencing: HiSeq 4000 (illumina); Density gradient fractionation system (Brandel, BR-188); Western blotting: Odyssey CLx system (LI-COR Biosciences); RT-qPCR: StepOnePlus Real-Time PCR System (Thermo Fisher Scientific, 4376600)
Data analysis	The computational pipeline that was used to analyze high-throughput DNA sequencing and RT-qPCR data is available on GitHub (https://github.com/YoichiroSugimoto/20211102_HP5_HIF_mTOR) and Zenodo (https://doi.org/10.5281/zenodo.6583247).<br><br>In the pipeline, the following software was used: R (4.0.0), data.table (1.12.8), dplyr (1.0.0), stringr (1.4.0), magrittr (1.5), ggplot2 (3.3.1), rcompanion (2.3.26), UMI-tools (1.0.1), Cutadapt (2.10), Bowtie2 (2.4.1), STAR (2.7.4a), paraclu (9), dpi (beta3), StringTie (2.1.2), ORFik (1.8.1), ViennaRNA Package (for RNALfold, 2.3.3), gprofiler2 (0.1.9), DESeq2 (1.28.0), DRIMSeq (1.16.0), apeglm (1.10.0), stageR (1.10.0), DEXSeq (1.34.0), mgcv (1.8-31), MACS2 (2.2.7.1), and DiffBind (2.16.0).<br><br>For RT-qPCR data collection, StepOne Software (2.3) was used. For western blotting data analysis, Image Studio (5.2) was used.

For manuscripts utilizing custom algorithms or software that are central to the research but not yet described in published literature, software must be made available to editors and reviewers. We strongly encourage code deposition in a community repository (e.g. GitHub). See the Nature Research guidelines for submitting code & software for further information.

Data

Policy information about availability of data

All manuscripts must include a data availability statement. This statement should provide the following information, where applicable:

- Accession codes, unique identifiers, or web links for publicly available datasets
- A list of figures that have associated raw data
- A description of any restrictions on data availability

The HP5 and 5' end-Seq of total mRNA data are available on ArrayExpress with the accession numbers E-MTAB-10689 and E-MTAB-10688 respectively. The Ct values for RT-qPCR analysis that were analyzed for Extended Data Fig. 3a are provided as Source data. mTOR hypersensitive genes identified by previous studies are provided as Source data. The ChIP-Seq data from Smythies et al. that were analyzed for Fig. 5d are available on GEO with the accession number GSE120885. The following reference data were used for this study; human genome: hg38, obtained via BSgenome.Hsapiens.UCSC.hg38 (1.4.3); human transcripts: RefSeq57 (GRCh38.p13) and GENCODE58 (GENCODE version 34: gencode.v34.annotation.gtf). Processed data files are provided as Supplementary Data and Source Data. The list of samples that were analyzed for this study is provided as Supplementary Data.

Field-specific reporting

Please select the one below that is the best fit for your research. If you are not sure, read the appropriate sections before making your selection.

☒ Life sciences ☐ Behavioural & social sciences ☐ Ecological, evolutionary & environmental sciences

For a reference copy of the document with all sections, see nature.com/documents/nr-reporting-summary-flat.pdf

Life sciences study design

All studies must disclose on these points even when the disclosure is negative.

Sample size	No statistical method was employed to predetermine sample size. The sample sizes (numbers of repetitions) were determined based on previous publications that used similar methodologies.
Data exclusions	Samples that failed to produce high-throughput DNA sequencing libraries within a multiplexed reaction (defined as < 25% of median number of reads) were excluded. Although this criterion was not pre-established, such samples were clearly outlying, implying technical failure. Other data exclusions are described in the manuscript.
Replication	The reproducibility of findings was assessed through biological replicate experiments. Only individual experiments using different clones derived from same cell line were treated as biological replicates. High-throughput DNA sequencing and western blotting experiments were performed with 2-4 biological replicates as reported in the figure legends or Supplementary Note. RT-qPCR was performed with 2 technical replicates since the purpose of the experiments was to show that the HP5 method has an equivalent quantitative power to RT-qPCR, and not to test a biological hypothesis. All attempts at replication were successful.
Randomization	Randomization is not relevant to this study because the samples were not allocated into separate experimental groups.
Blinding	Investigator was not blinded to group allocation. However, all the samples were processed and analyzed in the same/parallel manner, and the analyses were internally controlled (i.e. not subject to the bias of the investigator).

Reporting for specific materials, systems and methods

We require information from authors about some types of materials, experimental systems and methods used in many studies. Here, indicate whether each material, system or method listed is relevant to your study. If you are not sure if a list item applies to your research, read the appropriate section before selecting a response.

Materials & experimental systems

n/a	Involved in the study	
☐	☒	Antibodies
☐	☒	Eukaryotic cell lines
☒	☐	Palaeontology and archaeology
☒	☐	Animals and other organisms
☒	☐	Human research participants
☒	☐	Clinical data
☒	☐	Dual use research of concern

Methods

n/a	Involved in the study	
☐	☒	ChIP-seq
☒	☐	Flow cytometry
☒	☐	MRI-based neuroimaging

Antibodies

Antibodies used	(Primary antibodies; used at 1/1,000 dilution) anti-VHL (Santa Cruz Biotechnology, sc-135657, clone: VHL40), anti-HIF1A (BD

Antibodies used	Biosciences, 610959, clone: 54), anti-HIF2A (Cell Signaling Technology, #7096, clone: D9E3), anti-HIF1B (Cell Signaling Technology, #5537, clone: D28F3), anti-EIF4E2 (Proteintech, 12227-1-AP, polyclonal), anti-NDRG1 (Cell Signaling Technology, #9485, clone: D8G9), anti-SLC2A1 (Cell Signaling Technology, #12939, clone: D3J3A), anti-EGFR (Santa Cruz Biotechnology, sc-373746, clone: A-10), and anti-CA9 (Cell Signaling Technology, #5649, clone: D47G3); (secondary antibodies; used at 1/15,000 dilution) anti-mouse IgG DyLight 800 (Cell Signaling Technology, #5257) anti-mouse IgG IRDye 680RD (LI-COR Biosciences, 925-68072), and anti-Rabbit IgG IRDye 800CW (LI-COR Biosciences, 926-32213).
Validation	Primary antibodies that were used for this study are supported for use in immunoblotting of human proteins as follows.<br><br>Antibodies supplied by Cell Signaling Technology (anti-HIF2A, anti-HIF1B, anti-NDRG1, anti-SLC2A1, and anti-CA9 antibody) were validated for the recommended applications by the company as follows: (https://www.cellsignal.co.uk/about-us/cst-antibody-validation-principles). Anti-HIF1A antibody was validated for the immunoblotting of the human HIF1A according to the manufacturer (BD Biosciences). Anti-EGFR antibody was validated for the immunoblotting of human EGFR using a siRNA targeting EGFR in A-431 cells according to the manufacturer's website (Santa Cruz Biotechnology).<br><br>In addition, anti-VHL, anti-HIF1A, and anti-HIF2A antibody were validated by the comparison of human VHL-defective kidney cancer cells (RCC4 and 786-O cells) against cell lines with human VHL reintroduction, using immunoblotting. The validation experiments were performed using established cell lines produced in the laboratory, and consistent results were obtained for the cell lines generated in this study (Extended Data Fig. 2).<br><br>The antibodies for well established HIF target genes (NDRG1 and SLC2A1) were validated by comparing human VHL-defective kidney cancer cells (RCC4 and 786-O cells) with those with VHL reintroduced, using immunoblotting (Extended Data Fig. 6d).<br><br>Anti-EIF4E2 antibody was validated by comparing multiple clones of 786-O cells with intact EIF4E2 with those with CRISPR/Cas9 mediated inactivation of EIF4E2 (which was independently confirmed by the sequencing of the target genomic region.)

Eukaryotic cell lines

Policy information about cell lines

Cell line source(s)	RCC4 and 786-O cells were obtained from the Cell Services at the Francis Crick Institute.
Authentication	RCC4 and 786-O cells were authenticated by STR profiling by the Cell Services at the Francis Crick Institute.
Mycoplasma contamination	Cells were confirmed to be free from mycoplasma contamination by the Cell Services at the Francis Crick Institute.
Commonly misidentified lines<br>(See ICLAC register)	Both RCC4 and 786-O cells used for this study were not included in the list.

ChIP-seq

Data deposition

☒ Confirm that both raw and final processed data have been deposited in a public database such as GEO.

☒ Confirm that you have deposited or provided access to graph files (e.g. BED files) for the called peaks.

Data access links<br>*May remain private before publication.*	Published ChIP-Seq data (GEO accession ID of GSE120885) were re-analyzed and the experimental procedure is described in the original article (Smythies et al., EMBO Rep (2019)20:e46401).<br>The reprocessed peak data are available on GitHub (https://github.com/YoichiroSugimoto/20211102_HP5_HIF_mTOR).
Files in database submission	Raw data:<br>GSM3417826 RCC4_Normoxia_HIF-1a (PM14)_Rep 1<br>GSM3417827 RCC4_Normoxia_HIF-1a (PM14)_Rep 2<br>GSM3417828 RCC4_Normoxia_HIF-2a (PM9)_Rep 1<br>GSM3417829 RCC4_Normoxia_HIF-2a (PM9)_Rep 2<br>GSM3417830 RCC4_Normoxia_Input_Rep 1<br>GSM3417831 RCC4_Normoxia_Input_Rep 2<br><br>Processed data:<br>HIF-binding-site.bed
Genome browser session<br>(e.g. UCSC)	The peak data in the bed format are available on GitHub (https://github.com/YoichiroSugimoto/20211102_HP5_HIF_mTOR).

Methodology

Replicates	Duplicate experiments were performed for HIF1A, HIF2A, and input ChIP-Seq experiments (Smythies et al., EMBO Rep (2019)20:e46401)
Sequencing depth	All the sequence data are paired-end with the read length of 75 bases. The followings are the sequence depth:<br>RCC4 Normoxia HIF1A Rep 1: (total) 27493998 (uniquely aligned) 21955115<br>RCC4 Normoxia HIF1A Rep 2: (total) 27838028 (uniquely aligned) 22291592<br>RCC4 Normoxia HIF2A Rep 1: (total) 29535774 (uniquely aligned) 23752981

RCC4 Normoxia HIF2A Rep 2: (total) 29807598 (uniquely aligned) 23972781
RCC4 Normoxia input Rep 1: (total) 38705153 (uniquely aligned) 31431115
RCC4 Normoxia input Rep 2: (total) 42199628 (uniquely aligned) 34381727

Antibodies	Anti-HIF1A rabbit polyclonal, PM14 and anti-HIF-2α rabbit polyclonal, PM9. (Smythies et al., EMBO Rep (2019)20:e46401). The procedure to raise these two antibodies is described by Lau et al. (Br J Cancer. 2007 Apr 23; 96(8): 1284–1292.).
Peak calling parameters	The intersection of ChIP-Seq peaks identified by the ENCODE ChIP-Seq pipeline and MACS2 software was used. The ENCODE ChIP-Seq pipeline was used with the default parameters while the MACS2 was used with the following parameters (-q 0.1 --call-summits)
Data quality	The high quality of the data was demonstrated in the original study by Smythies et al. (EMBO Rep (2019)20:e46401).
Software	The Bowtie2, ENCODE ChIP-Seq pipeline (https://github.com/ENCODE-DCC/chip-seq-pipeline2) and MACS2 software. The pipeline to process the ChIP-Seq data is available on GitHub (https://github.com/YoichiroSugimoto/20211102_HP5_HIF_mTOR).
