# Supplementary figures and images for: Isoform-resolved mRNA profiling of ribosome load defines interplay of HIF and mTOR dysregulation in kidney cancer
Source: Nat Struct Mol Biol. 2022 Sep 12;29(9):871–80. doi: 10.1038/s41594-022-00819-2 (PMC9507966; doi:10.1038/s41594-022-00819-2)

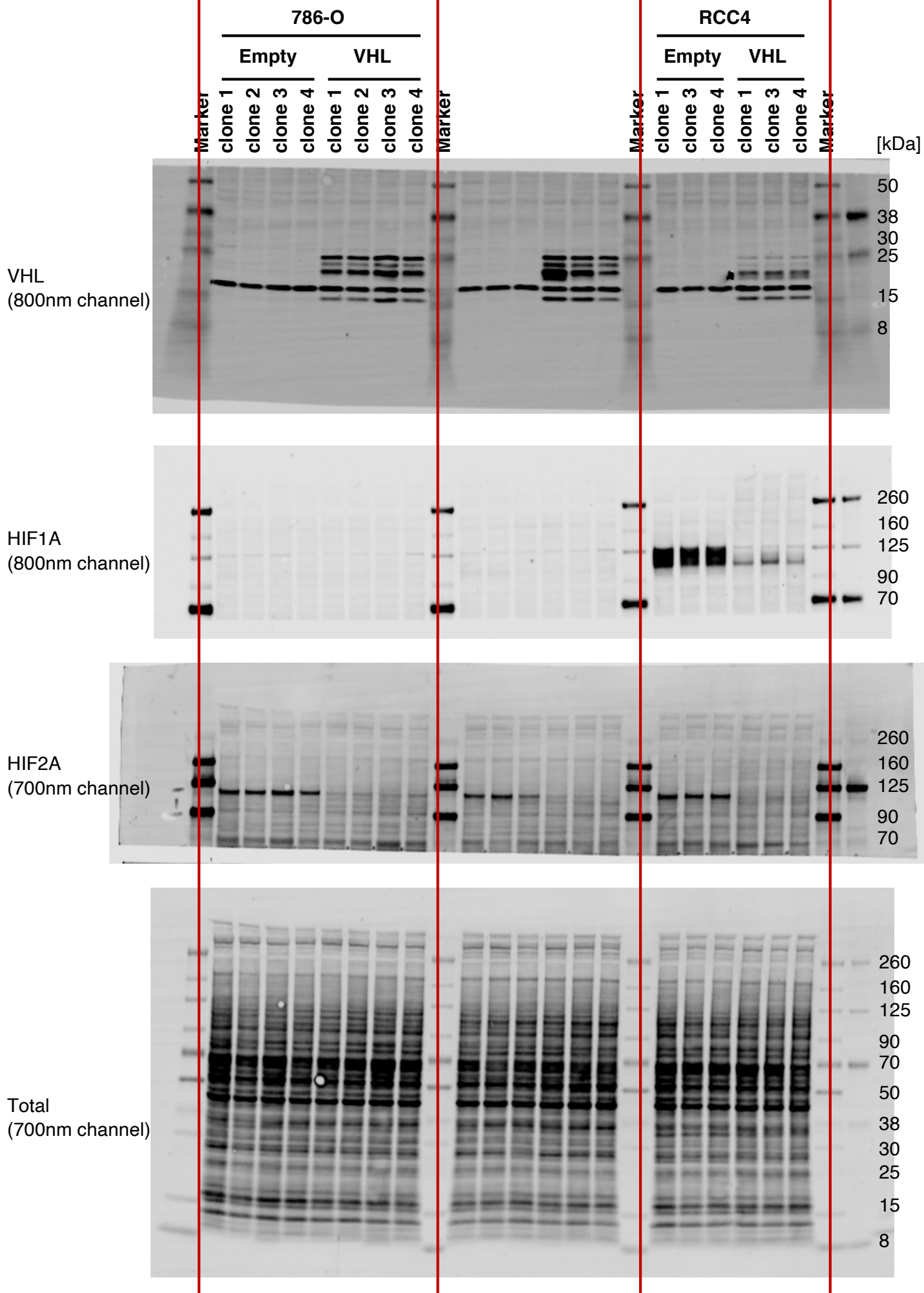

Supplement: Source Data Extended Data Fig. 2 — Unprocessed Blots [file 41594_2022_819_MOESM13_ESM.pdf]

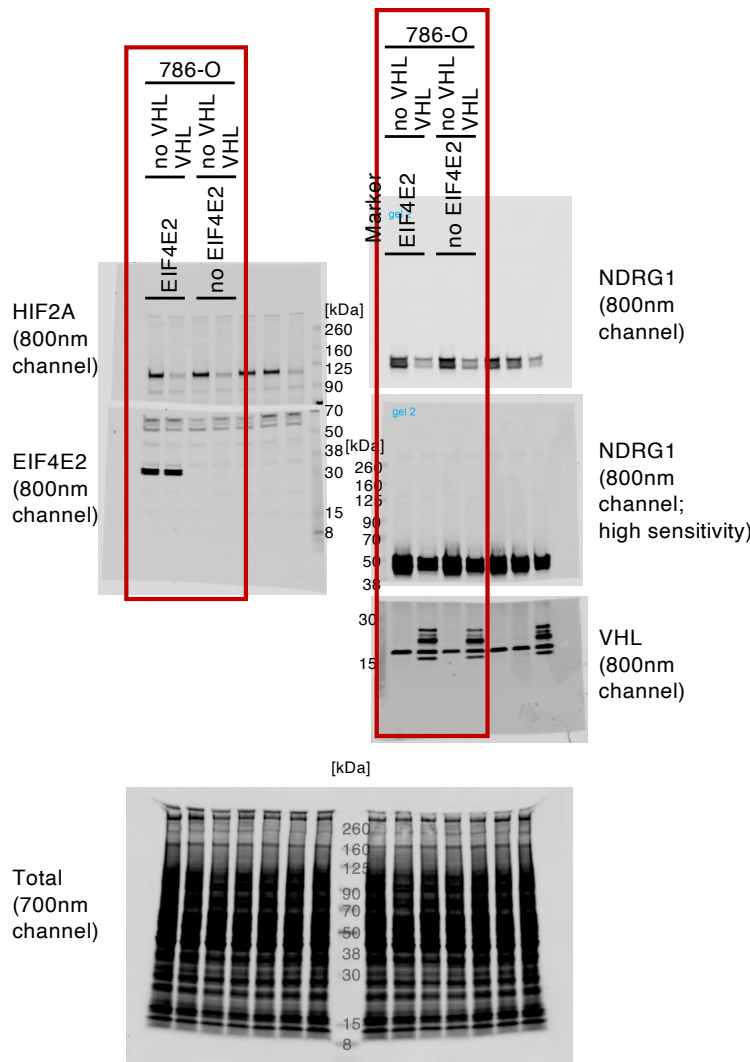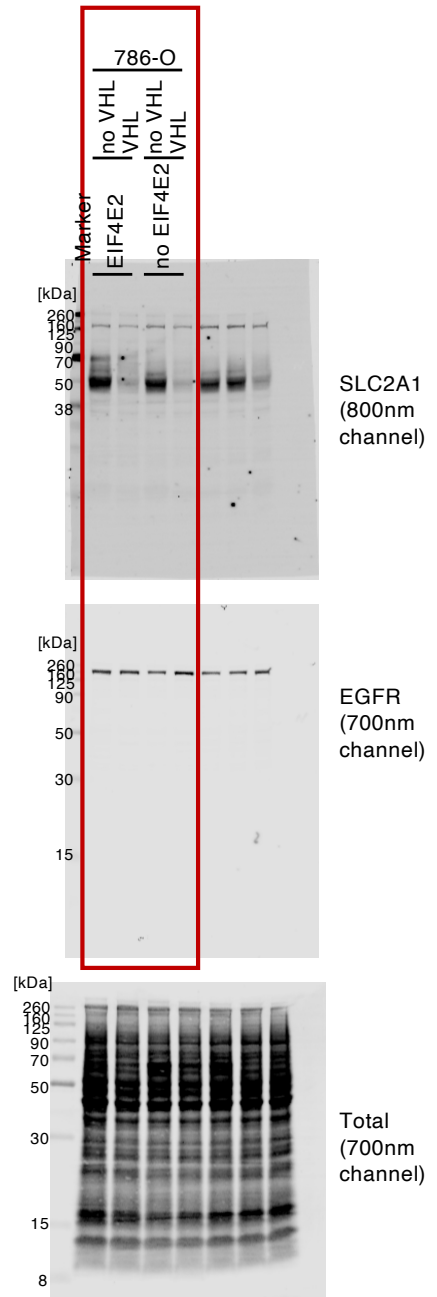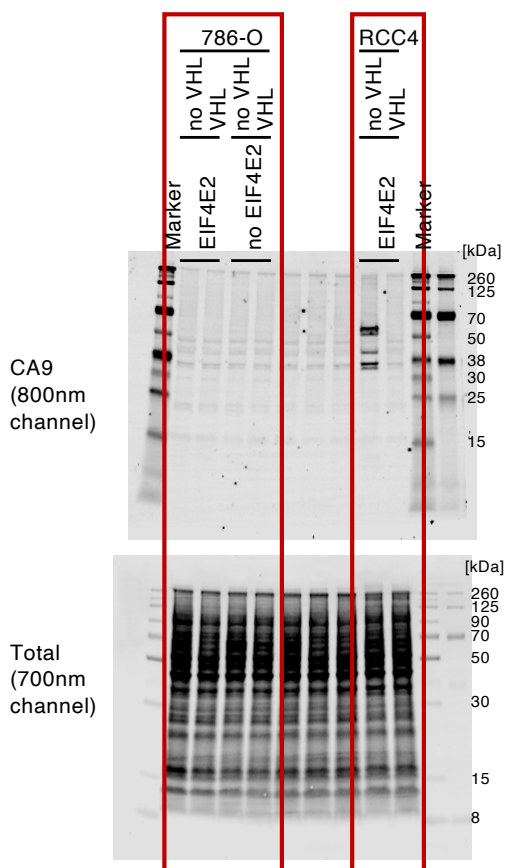

Supplement: Source Data Extended Data Fig. 6 — Unprocessed Blots [file 41594_2022_819_MOESM18_ESM.pdf]
